# Improving the Efficiency of New Automatic Dishwashing Detergent Formulation by Addition of Thermostable Lipase, Protease and Amylase

**DOI:** 10.3390/molecules22091577

**Published:** 2017-09-19

**Authors:** Ashwini Naganthran, Malihe Masomian, Raja Noor Zaliha Raja Abd. Rahman, Mohd Shukuri Mohamad Ali, Hisham Mohd Nooh

**Affiliations:** 1 Enzyme and Microbial Technology Research Center, Universiti Putra Malaysia, Serdang, Selangor 43400, Malaysia; ashwininaganthran@gmail.com (A.N.); masomian2000@yahoo.com (M.M.); mshukuri@upm.edu.my (M.S.M.A.); more.knock@gmail.com (H.M.N.); 2 Department of Microbiology, Faculty of Biotechnology and Biomolecular Science, Universiti Putra Malaysia, Serdang, Selangor 43400, Malaysia; 3 Department of Biochemistry, Faculty of Biotechnology and Biomolecular Science, Universiti Putra Malaysia, Serdang, Selangor 43400, Malaysia; 4 Laboratory of Molecular Biomedicine, Institute of Bioscience, Universiti Putra Malaysia, Serdang, Selangor 43400, Malaysia

**Keywords:** detergent, dishwashing, lipase, amylase, protease, thermostable enzyme, soil removal, hard water

## Abstract

The use of T1 lipase in automatic dishwashing detergent (ADD) is well established, but efficiency in hard water is very low. A new enzymatic environmentally-friendly dishwashing was formulated to be efficient in both soft and hard water. Thermostable enzymes such as T1 lipase from *Geobacillus* strain T1, Rand protease from *Bacillus subtilis* strain Rand, and Maltogenic amylase from *Geobacillus* sp. SK70 were produced and evaluated for an automatic dishwashing detergent formulation. The components of the new ADD were optimized for compatibility with these three enzymes. In compatibility tests of the enzymes with different components, several criteria were considered. The enzymes were mostly stable in non-ionic surfactants, especially polyhydric alcohols, Glucopon UP 600, and in a mixture of sodium carbonate and glycine (30:70) buffer at a pH of 9.25. Sodium polyacrylate and sodium citrate were used in the ADD formulation as a dispersing agent and a builder, respectively. Dishwashing performance of the formulated ADDs was evaluated in terms of percent of soil removed using the Leenert‘s Improved Detergency Tester. The results showed that the combination of different hydrolysis enzymes could improve the washing efficiency of formulated ADD compared to the commercial ADD “Finish” at 40 and 50 C.

## 1. Introduction

Detergent is a substance containing a certain chemical that can remove dirt. Nowadays, detergents consist of highly-developed surfactants and water softeners. These detergents are superior to soaps because they can perform better washing in the presence of metallic ions such as Ca^2+^ and Mg^2+^ than normal soap. Sodium tripolyphosphate is a popular surfactant because of its high chelating property. Chlorines, on the other hand, improve washing via active oxygen species in redox reactions. Unfortunately, increasing use of these two chemicals has caused ecological issues when they enter water streams. Phosphates cause massive algal growth (eutrophication), while chlorines kill fish, disturbing the ecosystem’s equilibrium [1].

Due to these ecological issues, modern detergents have moved from being petrochemical-based to oleochemical-based to be more environmentally friendly. Consequently, enzymes have been introduced as additives in detergent formulations. The development of enzymes such as lipases and adding them into the detergent mix was motivated by the need to save energy by washing in lower temperatures and by the environmental concerns of reducing pollution by non-biodegradable chemicals, as well as the success of companies such as Novo Industry A/S in creating novel enzymes [2]. Lipase is added to break down insoluble triglycerides into fatty acids—which have a lower surface tension—creating even smaller emulsion for easy removal [3]. However, scientists have found that the incorporation of more than one enzyme increases washing efficacy. Among these enzymes, cellulase can enhance fabric appearance as well as featuring softening, soil removal and color brightening [4]. Proteases aid the removal of proteinaceous food stains whereas amylases remove starch based stains [5]. Enzymes are better than conventional chemicals, as they can get rid of stubborn stains but degrade before entering waterways, alleviating or preventing water pollution problems. ADD consists of basic components such as surfactants, bleaches, water softeners and auxiliary agents. However, these basic components do not provide good cleaning efficacy therefore other components need to be added to the formulation to improve washing efficacy. They usually contain low foaming surfactants, because foams reduce water pressure and washing efficiency [6]. Their performance is evaluated in terms of cleaning power and Detergent Biodegradability. A good dishwasher detergent must reduce surface tension to allow easy removal of soils, work efficiently even in hard water, minimize water spots, and reduce glass and metal corrosion due to heat [7]. Therefore, a comparable environmentally-friendly and multipurpose ADD formulation is necessary to meet industrial demand.

Previously, Rahman et al. [3] developed an ADD formulation containing only T1 lipase. This formulation showed only 40% of soil removal in hard water. In order to improve upon that formulation, another two different thermostable enzymes, Rand protease and Maltogenic amylase, were added along with some modifications on the formulation of the previous ADD. The aim of this study was to improve the efficacy of washing, addressing dirt from the three substrate categories—starchy food, protein stains and fatty stains. Therefore, detergent formulations containing all three enzymes and compatible detergent components were evaluated for dishwashing performance under selected conditions.

## 2. Results and Discussion

### 2.1. Stability of T1 Lipase, Rand Protease and Maltogenic Amylase In Detergent Components

With the same protein concentrations of T1 lipase, Rand protease and Maltogenic amylase, 15, 100 and 230 U/mL enzyme activities were found and referred to as 1X, 1Y and 1Z, respectively. This unit of enzymes has been used for the optimization of all detergent components, including surfactants, bleaches, builders, dispersing agents and alkalinity agents. The stability of T1 lipase, Rand protease and Maltogenic amylase were checked via a compatibility test. The results are shown in Table 1.

The results show that non-ionic surfactants were compatible for all three enzymes except SDS, a cationic surfactant, which destabilized all three enzymes. T1 lipase was most stable in the presence of G600, followed by Tween 80, PEG300. Sugar alcohol could lessen the thermal denaturation and allow an enzyme to react and release more products, which explains the over-activity of T1 lipase in the presence of G600 [3]. T1 lipase was slightly inhibited (15%) by Tween 80 and PEG300. Rand protease was very stable in both Tween 80 and G600. Tween 80 activated Rand protease, that might be due to the unfolding of the substrate moiety by surfactant [8]. The relative activity of Maltogenic amylase was increased by G600, followed by PEG 300. Also, a decrease of less than 7% was observed in the relative activity of Maltogenic amylase in the presence of Tween 80. Overall, G600 was the best surfactant for all three enzymes, since it stabilized them. Similarly, von Rybinski and Hill [9] reported on the stability of lipase, protease, amylase and cellulase in the presence of alkyl polyglucoside, G600. SDS is an anionic surfactant which is well known in detergent formulation. However, SDS destabilized the T1 lipase, Rand protease and Maltogenic amylase tested in this study. The ionic interactions between enzymes and the SDS head group may cause the inactivation of globular proteins [10].

Bleaching agents, sodium percarbonate, and sodium perborate destabilized all three enzymes (Table 1). The bleaching agents released hydrogen peroxide—an oxidizing agent—when dissolved into water. This could easily lead to the oxidization of some amino acids and cause the inactivation of enzymes. Most commercial proteases have been reported to be unstable in the presence of bleaching agents [11]. However, Maxamal is a genetically modified protease produced by mutagenesis that has shown improved stability in the presence of bleaching agents [12]. Other examples of oxidative stable mutants enzymes include Durazym, Purafect [11] and Lipolase^®^ [12].

Sodium polyacrylate was the only dispersing agent tested in this study (Table 1). The activity of T1 lipase and Rand protease were moderately affected by polyacrylate, whereas Maltogenic amylase showed more than 70% stability compared to the control.

The builders tested in this study had a different impact on the stability of the enzymes. In the presence of sodium citrate, T1 lipase and Rand protease were moderately stable and Maltogenic amylase retained 96% of its stability. However, sodium metasilicate and sodium silicate destabilized all three enzymes. Since T1 lipase and Rand protease are Ca^2+^ dependent enzymes, this builder likely competes with the enzymes for the available Ca^2+^ ions, which can contribute to effect on the stability of enzymes [13].

A compatibility test of T1 lipase, Rand protease, and Maltogenic amylase with different alkalinity agents is presented in Table 1. Rand protease was reported to have a broad range of pH stability of between pH 6.5 to 10.0 [14], whereas T1 lipase was reported to be stable between pH 6.0 to 11.0 [15] and Maltogenic amylase was found to be stable in the range of pH 6.0 to 9.0 [16]. Since they have similar ranges, it is easier to find suitable alkalinity agents for them. T1 lipase has an optimum pH of 9.0, whereas Rand protease and Maltogenic amylase have an optimum pH of 7.0. All three enzymes showed high stability in Sodium carbonate-glycine, at a pH of 9.25. However, the mixture of SC: SB at pH 9.5 drastically decreased the activity of T1 lipase and also had a moderate effect on Rand protease and Maltogenic amylase which indicated that the enzymes are affected by the component of the buffer, not only the pH.

Water hardness can be reduced, as most alkalinity agents tend to bind to cations. Phosphate pH 7.0 and Sodium bicarbonate pH 8.6 reduced the relative activity of T1 lipase. This might be due to the binding of phosphate and carbonate to Ca^2+^, producing Ca_3_(PO_4_)_2_ and CaCO_3_ precipitates [17], while Ca^2+^ is an essential ion for T1 lipase structural stability [15]. Rand protease was found to be stable in Phosphate and Tris-HCl buffers, pH 7.0. However, Tris-HCl buffer at a pH of 7.0 decreased the activity of T1 lipase and Maltogenic amylase. Sodium bicarbonate, pH 8.6, increased the activity of Maltogenic amylase much higher than did the other buffers tested in this study, indicating that the CaCO_3_ precipitates could help stabilize the enzyme. Sulong [16] reported a drastic inhibition of the activity of Maltogenic amylase in the presence of Ca^2+^ ions in the reaction mixture.

In summary, Glucopon UP 600 as surfactant, sodium polyacrylate as the dispersing agent, sodium citrate as builder, and Sodium carbonate–glycine (30:70), pH 9.25, as buffer were used in a new ADD formulation. None of the bleaching agents were added to the formulation, which makes the new ADD more environmentally friendly. Bleaching agents are usually used to break down stains and kill bacteria, both of which can be done using enzymes. Moreover, using the new ADD, washing will be performed at high temperatures, as hot water can kill most bacteria.

### 2.2. Efficiency of Detergent Formulation

**Figure 1:**
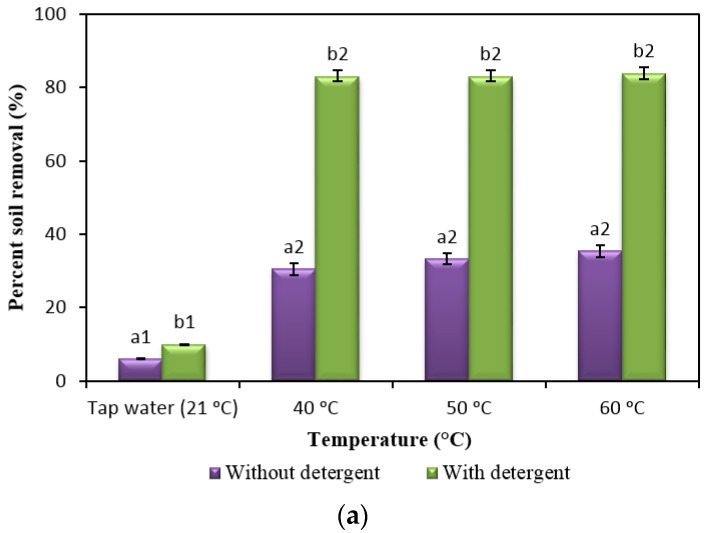

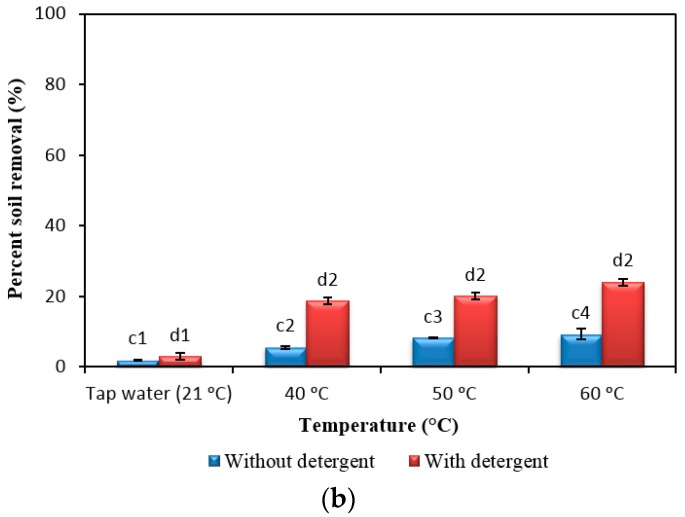
Efficiency of formulated detergent. The detergent contained 7% surfactant, 3% builders, 5% dispersing agent and was buffered with glycine-carbonate (pH 9.25) and tested in (**a**) Soft water (0 ppm CaCO_3_) and (**b**) Hard water (350 ppm CaCO_3_) at 40 °C, 50 °C and 60 °C. Tap water (21 °C) used as a control. Note: Superscript a2, b2, c2, c3, c4 and d2 indicated groups that showed a significant difference between their respective groups. All superscripts were obtained using post-hoc tests as shown in Table S1. Data are means ± standard deviation of three determinations and indicated as an error bar.

The efficiency of detergent formulated without enzymes in different temperatures at 0 ppm CaCO_3_, soft water (Figure 1a), and 350 ppm CaCO_3_, hard water (Figure 1b), has been studied to measure the washing efficacy of the newly formulated detergent before the addition of enzymes.

The addition of detergent increased the dishwashing performance at 0 ppm up to 350 ppm compared to the control (without detergent). This improvement is due to the components present in the detergent. The presence of a surfactant can reduce surface tension and ease the removal of soil, while a dispersing agent can prevent the removal of soil from the surface and a builder can reduce the hard water effect. Based on Figure 1, even though washing performance in hard water increased as temperature increased, the washing performance in soft water showed a better result in the presence of detergent. This might be due to the presence of Ca^2+^ and Mg^2+^ ions in the hard water, which can affect the ionic surfactant and subsequently form a highly charged structure, preventing soil removal from the hard surface [3].

### 2.3. Efficiency of Individual Enzyme Concentration

In the next experiment, the soil removal performance of the formulated detergent with the addition of individual free enzymes at different concentration was investigated to determine the best amount of lipase, protease and amylase required in the formulation (Figures S1 and S2).

A different amount of each enzyme was individually added into the soft and hard water at 60 °C. The results showed that the high concentration of T1 lipase, Rand protease and Maltogenic amylase did not substantially improve washing. Based on post-hoc analysis, there was no signification difference in the lowest amount of enzyme compared to other amounts in soft water (Table S2). However, in hard water, post-hoc analysis showed significant differences between 1% and the higher amounts of T1 lipase, between 3% and 6% and the higher amount of Rand protease and between 1% and the higher amounts of Maltogenic amylase (Table S3). Therefore, 3% of T1 lipase, 9% of Rand protease and 1.5% of Maltogenic amylase were incorporated together into the same detergent.

The lowest possible enzyme level with good efficiency used in the detergent formulation is considered economical for future application. Moreover, a study by Hemachander and Puvanakrishnan [18] showed that the addition of lipase into protease-containing detergents improved the washing efficiency and removal of fatty stains, demonstrating that combinations of more than two enzymes may be a better option.

### 2.4. Enzymes Encapsulation Performance

Encapsulation of T1 lipase using additives, gum arabic (GA) and maltodextrin (MD) resulted in activity retained compared to the free enzyme (Table 2). The activity loss was greater in the powder form of the enzyme; the spray dried supernatant containing 3% (*w*/*v*) T1 lipase without any wall material, compared to the encapsulated or free enzyme—which is the control—before spray drying. The loss of activity in the powder form of the enzyme may be due to the thermal denaturation of the enzyme during the spray drying process.

However, the addition of additives had a positive effect on the enzyme and led to an increase in the total activity. This suggests that the additives protect the enzymes from direct exposure to the spray dryer heat. In addition, it also reduces the stickiness and hygroscopicity of the powder [19]. Hygroscopicity refers to the ability to absorb moisture from the air.

**Figure 2:**
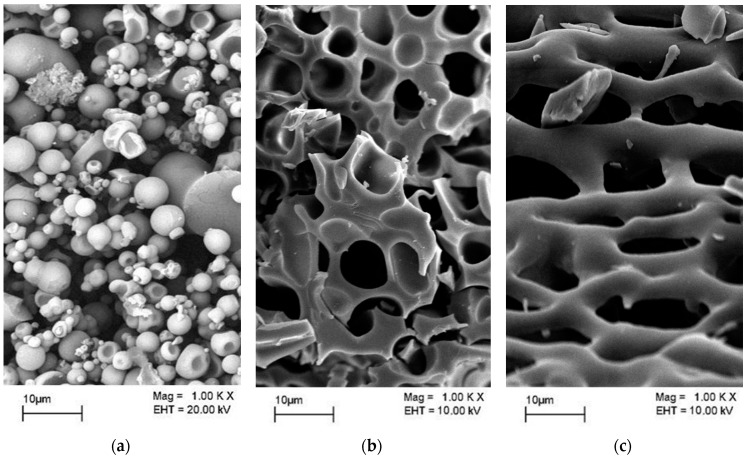
Scanning Electron Microscopy of the immobilized enzymes. (**a**) SEM of the spray-dried powder of T1 lipase; (**b**) SEM of the freeze-died powder of Rand Protease; (**c**) SEM of the freeze-died powder of Maltogenic amylase.

The blend, consisting of GA/MD/T1 at a ratio of 6:12:3, produced products that were mostly spherical, with a smooth surface and few dents (Figure 2a). Moreover, the products from this blend also yielded a two times higher residual enzymatic activity than did the free enzyme (Table 2). The addition of wall materials also made spray-dried products more soluble compared with the free enzyme. This is because GA and MD are hydrophilic and soluble in water—however, GA is highly viscous upon dissolution in water, especially at concentrations exceeding 10% (*w*/*v*).

Although the spray-dried product from the blend of GA/MD/T1 yielded decent powder morphology and characteristics as well as satisfactory enzymatic activity retention, the size distribution of the powder was still wide, and many of the powder sizes were still less than 100 μm. At very low particle sizes, the enzyme powder may become a health risk. Therefore, agglomeration or granulation is typically preferable to spray-drying alone.

To prepare the powder form or to encapsulate the Rand protease and Maltogenic amylase, spray drying was performed because both enzymes are thermostable and able to survive at high temperatures. But, due to exposure of the enzymes to high temperatures of 85 °C, their residual activity was very low and almost denatured. Therefore, in the next attempt, the enzymes were freeze dried. The advantages of the enzyme freeze drying include high recoveries of the end products of organic analytes regardless of water content and the prevention of the volatilization of temperature-sensitive analytes. However, in the freeze drying process, using low temperatures to dry an enzyme can induce several stresses that may cause denaturation [20].

Based on Table 2, the enzyme activity of the powder forms of the Rand protease and Maltogenic amylase were lower than the free enzymes, but the results were better than spray drying the enzymes. Even though low temperatures in freeze-drying were generated for the drying process to take place, this can still deactivate and destabilize the enzymes due to dehydration stress [19]. Hence, stabilizers must be used when freeze-drying in order to decrease the deactivation and destabilization of the enzyme.

After the addition of additives to encapsulate the enzymes, the results showed that both encapsulated Rand protease and Maltogenic amylase produced higher activities compared to the powder form and free enzymes, at 93% and 95% of activity retained, respectively.

This proves that addition of additives to encapsulate the enzymes showed positive effects on both enzymes. A study explained that gum arabic and maltodextrin can act as effective coats during freeze drying for serine proteases [19]. Gum arabic decreases the hydrophobicity of the product and increases water mobility in the enzyme, whereas maltodextrin provides good oxidative stability to encapsulated enzymes. The dry matter of the powder form of Rand protease without the addition of gum arabic and maltodextrin was too low and sticky. This stickiness was due to residues of the nutrient medium [21]. A fluent powder was produced after the addition of gum arabic and maltodextrin.

The immobilization of protease (Figure 2b) and Maltogenic amylase (Figure 2c) was analyzed using scanning electron microscopy (SEM). The protease was immobilized with gum arabic and maltodextrin additives, whereas the amylase was immobilized with only gum arabic. The scan of the freeze-dried powders under the microscope showed a glass-like structure with a smooth surface. It was reported that during the dehydration process, an amorphous glassy structure was produced to protect the entrapped molecules from opening due to exposure to oxygen and heat [22].

### 2.5. Comparison of Detergent with Free and Encapsulated Enzymes

**Figure 3:**
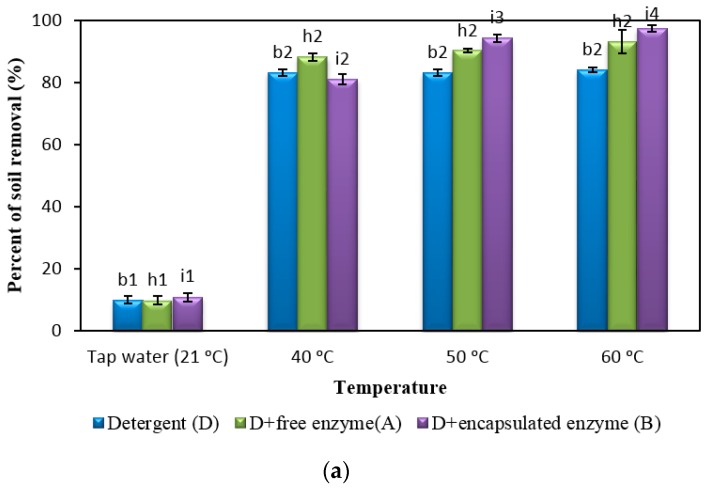

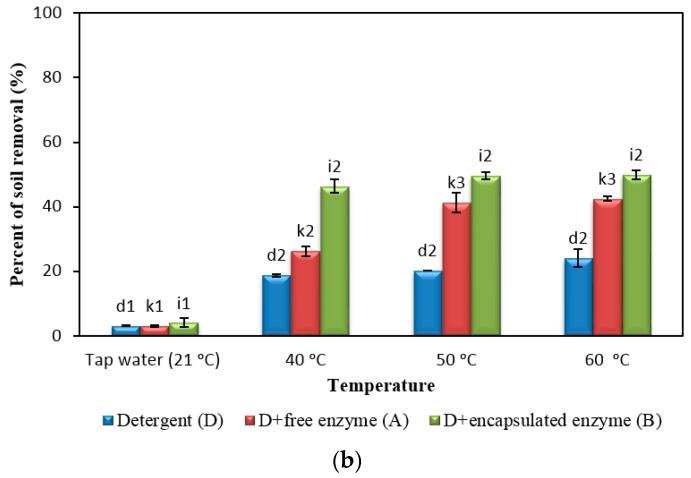
Dishwashing performances of the formulated detergent with free and encapsulated enzymes. The washing was conducted at different water temperatures and hardness. The formulated detergent mixture and enzymes, in (**a**) soft water (0 ppm CaCO_3_) and (**b**) hard water (350 ppm CaCO_3_) at 40, 50 and 60 °C. Tap water (21 °C) used as a control. Note: Superscripts b2, h2, i2, i3, i4, d2, k2, k3 and l2 indicated groups that showed a significant difference between the groups when washings were done. All superscripts were obtained using post-hoc tests as shown in Table S3. Data are means ± standard deviation of three determinations and indicated as error bars.

In the following experiment, the dishwashing efficiency of the formulated detergent after the addition of free and encapsulated enzymes was tested at different water temperatures (40, 50 and 60 °C) and hardness (Figure 3). Dishwashing temperatures were used. The water hardness used for dishwashing was 0 ppm CaCO_3_ for soft water (Figure 3a) and 350 ppm CaCO_3_ for hard water (Figure 3b). The detergent containing free enzymes was labeled Detergent A and the detergent containing encapsulated enzymes was labeled Detergent B.

As expected, dishwashing performance improved as the temperature increased for both formulated ADDs with enzymes. Based on Figure 3a, detergent without enzymes showed around 80% dishwashing performance from 40 to 60 °C in soft water. Meanwhile, both formulated detergents A and B showed no visible difference in dishwashing performance after the addition of enzymes at 21 °C in both soft and hard water. This proves that enzymes are not active at the lower temperature, hence no washing improvement was observed. There are studies that explain that some fats inhibit enzymes reaction at low temperature since they exhibit higher melting points [23]. In addition, T1 lipase relative activity was reported to decrease up to 75% as the temperature decreases from 70 to 40 °C, which explains the low dishwashing performance at 21 °C. A similar pattern was shown by native Maltogenic amylase, with relative activity decreasing at temperatures lower than 55 °C [16].

Based on post hoc studies, in soft water at 50 and 60 °C, the addition of the encapsulated enzyme caused a significant difference in detergent performance compared to the free enzyme. It can be concluded that in soft water encapsulated enzyme detergent may work better in high temperatures.

In hard water, by increasing the temperature, the dishwashing performance after the addition of encapsulated and free enzymes showed significant gains in efficiency compared to the detergent without enzyme. However, the performance of formulated detergents decreases in hard water compared to soft water in the same conditions. A few studies explain that the performance of surfactant can be severely affected by the presence of cations Ca^2+^ and Mg^2+^. These cations form highly charged structures with the surfactant, which prevent the removal of soil [24]. The results showed that incorporation of three encapsulated enzymes into the formulation increased the dishwashing performances when increasing the temperature up to 60 °C. The improvements of the dishwashing performance in both soft and hard water were more dramatic with the addition of encapsulated enzymes at 50 and 60 °C. This may be because both Maltogenic amylase and crude Rand protease work at an optimum temperature of 50 °C. Also, the majority of the detergents have high efficiency in soft water but fail to perform in hard water [25]. Hence, formulated detergent containing encapsulated enzymes with high efficiency in hard water could fulfill industrial demand.

In hard water, soil removal by Detergent A at 40 °C is lower compared to the higher temperature while this performance is almost same for Detergent B, which contained encapsulated enzymes. The better performance of Detergent A at a higher temperature could be due to the carbonate hardness of water , which can be reduced at higher temperatures [26]. The carbonate hardness has easier access to the free enzymes compared to the encapsulated enzymes.

Post hoc tests revealed that there is no significant difference between the dishwashing performance of detergents from 50 to 60 °C for both the encapsulated and free enzymes in hard water. Therefore, dishwashing performance is nearly the same. It can be concluded that optimum washing temperature for this detergent is in the range 50 to 60 °C.

### 2.6. Effect of Detergent Concentration on Removal of Soil

The effect of the detergent concentration on washing performances was studied. Table 3 shows the comparison of the dishwashing performance between Detergents A and B in different detergent concentrations with a constant amount of enzymes in hard water at 50 °C. Both formulated ADDs showed an increase in the percentage of soil removal with an increase of detergent concentration; however, the post hoc tests revealed no significant differences in performance between the 1.5, 2.0, and 2.5% of added detergent concentration, suggesting that the lowest amount of detergent would sufficiently show good dishwashing performance. Hence, in this study, 1.5% was found to be the ideal detergent concentration for soil removal. Chauhan and Garlapati [27] reported that the decreasing soil removal by increasing detergent concentration might cause the inhibition of enzymes at higher detergent concentrations.

### 2.7. Effect of Washing Time on Removal of Soil

**Figure 4:**
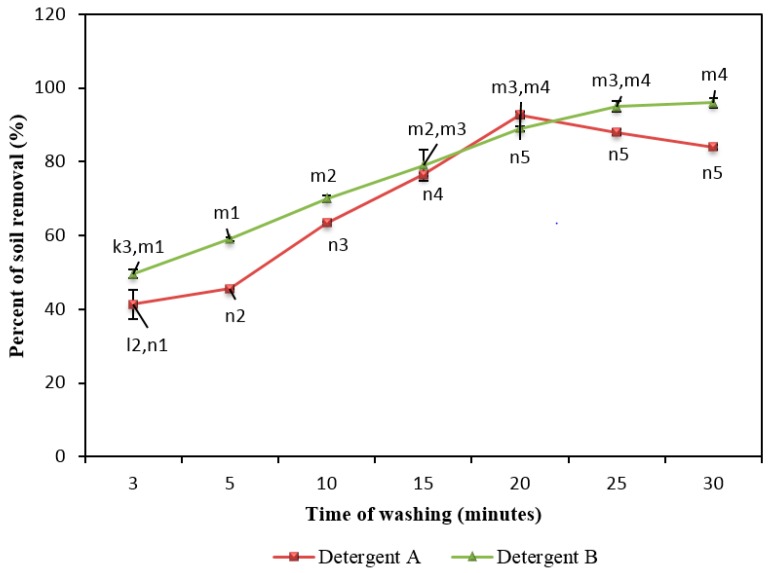
Effect of washing time on soil removal using the formulated detergent A (contained free enzymes) and B (contained encapsulated enzymes) in hard water (350 ppm CaCO_3_) at 50 °C. Note: Superscripts m2, m3 and m4 (test for detergent A) indicated groups that showed significant difference between the groups when washings were done. Superscripts n2, n3, n4 and n5 (test for detergent B) indicated groups that showed a significant difference between the groups when washings were done. All superscripts were obtained using post-hoc tests as shown in Table S4. Data are means ± standard deviation of three determinations and indicated as error bars.

The effect of washing time on soil removal was studied using Detergent A (free enzymes) and Detergent B (encapsulated enzymes) (Figure 4). The maximum dishwashing performances by Detergents A and B were achieved 92% and 96% at 20 and 30 min, respectively. Detergent A showed an increase of soil removal with an increase in dishwashing time from 3 to 20 min and decrease at 25 to 30 min. In contrast, Detergent B showed an increase of soil removal up to 30 min.

It was observed that the Detergent A worked properly for soil removal with a longer washing time up to minute 20. The longer washing time for Detergent A (up to 20 min) might be due to the high thermostability of the three incorporated enzymes. T1 lipase, Rand protease and Maltogenic amylase retained 100% of enzyme activity after 30 min incubation at 55 °C [14,28,29]. Hence, after 25 min, the redeposition of soil onto slides washed might take place. More soil can be removed as washing time increases, but this would reach a point at which the final trace of soil may never be removed as redeposition would occur as rapidly as soil removal. However, cold water prevents redeposition better than warm water [30]. In addition, free enzymes act faster towards the breakdown of soils because they target the substrate easily. But they denature easily, since they are exposed to denaturant. Detergent B showed an increasing dishwashing performance up to 20 min, but the post hoc test revealed that the slight increase in dishwashing performance at 25 or 30 min is not a significant improvement.

### 2.8. Comparison of Formulated Detergents Containing Enzymes with Commercial

**Figure 5:**
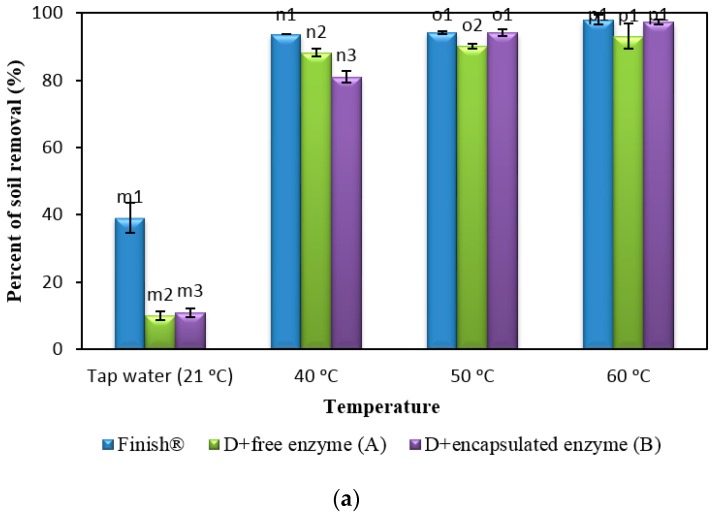

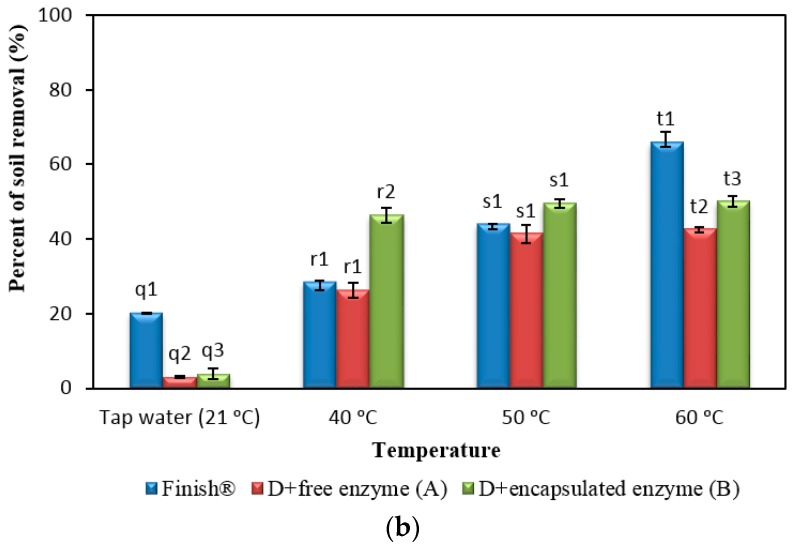
Comparison of dishwashing performance of the formulated detergents A (contained free enzymes) and B (contained encapsulated enzymes) with commercial ADD, Finish^®^ in (**a**) Soft water (0 ppm CaCO_3_) and (**b**) Hard water (350 ppm CaCO_3_) at 21 °C, 40 °C, 50 °C and 60 °C. Note: Superscripts m2 and m3 (for test in 21 °C soft water), n2 and n3 (for test in 40 °C soft water), o2 (for test in 50 °C soft water), q2 and q3 (for test in 21 °C hard water), r2 (for test in 40 °C hard water), t2 and t3 (for test in 60 °C hard water) showed significant difference between the groups of same water temperature. All superscripts were obtained using post-hoc tests as shown in Table S6. Data are means ± standard deviation of three determinations and indicated as error bars.

After evaluation of both formulated detergents in both soft and hard water at different temperatures, the formulated detergents with enzymes were compared with a commercial ADD, Finish^®^, as shown in Figure 5.

Detergent A showed comparable performances with Finish^®^ at 50 °C in 0 ppm CaCO_3_, soft water (Figure 5a). Detergent B showed a better washing efficiency in soft water at a higher temperature with significant differences (Figure 3a). Dishwashing performance using Detergent B at 50 and 60 °C in soft water showed no significant improvements—however, these performances are comparable to Finish^®^. This suggests that dishwashing using Detergent B can be done at a lower temperature to save electric current and the amount of water used in washing (http://www.indesitservice.co.uk/help-and-advice/dishwasher/).

The dishwashing efficiency of formulated detergent with a commercial detergent in hard water was compared and is presented in Figure 5b. The efficiency of Detergent B is better than Detergent A and Finish^®^ at 40 °C in hard water. In hard water, both formulated detergents were as efficient as Finish^®^ at 50 °C, with no significant difference in washing. At 60 °C, in hard water, post hoc tests revealed significant differences between Detergent A, B and Finish^®^. Finish^®^ showed better dishwashing performance, followed by Detergent B and Detergent A. Dishwashing at 60 °C in 350 ppm of CaCO_3_ with Detergent A, B and Finish^®^ resulted in 42.5%, 50% and 66% soil removal, respectively. Also, among the other tested temperatures in this study, Finish^®^ showed superior soil removal performance at 60 °C, which has been reported as the optimal operating temperature for commercial dishwashing.

The formulated detergent containing triple enzymes showed better soil removal than the detergent containing only T1 lipase. Rahman et al. [3] reported that the formulated detergent containing only T1 lipase showed the maximum washing of 30% at 60 °C in the same condition. This explained that low dishwashing performance was due to the nature of the soil used, which contained fat, proteins, and carbohydrates. Hence, incorporation of three different thermostable hydrolysis enzymes into the formulation increased dishwashing performance.

It is concluded that Detergents A and B showed the best performance in both soft and hard water at temperatures of 50 and 60 °C. However, this would not really be of benefit in most situations, since the same performance can be achieved at a lower temperature to reduce electricity usage and the amount of water used in washing. Thus, the best energy-saving working temperature of these ADDs is 50 °C.

## 3. Materials and Methods

### 3.1. Materials

All chemicals and solutions were used with standard abbreviations and acronyms of chemical compounds. Ampicillin and chloramphenicol were obtained from Calbiochem, USA. Isopropyl β-d-1-thiogalactopyranoside (IPTG) was purchased from 5-Prime, Hamburg, Germany. Virgin olive oil (Bertolli, Italy) and Skippy creamy peanut butter (Unilever, Lumpur, Malaysia) were supplied from a local supermarket. All other chemicals used in this study were analytical grade from Sigma-Aldrich and Merck.

### 3.2. Enzyme Production

#### 3.2.1. T1 Lipase

T1 lipase was produced based on the method described by Rahman et al. [3]. The T1 lipase protein was expressed in *E. coli* BL21 harboring a T1 gene. The bacteria were grown in 200 mL LB broth containing 50 µg/mL ampicillin and 35µg/mL chloramphenicol. The culture was induced using 0.025 mM IPTG when OD_600nm_ reached 0.75 and was incubated for 12 h at 37 °C. Then, the culture was centrifuged at 10,000× *g* for 10 min at 4 °C. The pellet was resuspended in 50 mM Glycine-NaOH, pH 9.0 and sonicated for 4 min inclusive of the interval time. The intercellular crude T1 lipase (free enzyme) was obtained by centrifuging the sonicated cells at 10,000× *g* for 15 min at 4 °C. To prepared the powder form of T1 lipase, the free enzyme was spray dried using BÜCHI mini spray dryer B-290 (St. Gallen, Switzerland) at inlet 160 °C, outlet 85 °C, feed rate of 25 mL/min, and an air flow rate of 536 L/h.

#### 3.2.2. Rand Protease

Rand protease was produced using a method described by Abusham et al. [14]. The *Bacillus subtilis* bacterium was grown in 10 mL nutrient broth (NB) at 37 °C with 200 rpm agitation rate. Then, the culture was centrifuged at 4000× *g* for 5 min at 4 °C. To prepare the inoculum, the pellet was resuspended in sterile 0.85% (*w*/*v*) NaCl solution until the OD_600nm_ reached 0.5. Then, 5% (*v*/*v*) of inoculum was transferred into 100 mL production medium and incubated at 37 °C for 20 h. The extracellular crude Rand protease (free enzyme) was obtained by centrifuging the culture at 12,000× *g* for 10 min at 4 °C. The powder form was obtained by freeze drying the free enzyme at −45 °C for 48 h until it dried completely using Alpha 1-2 LD plus freeze dryer.

#### 3.2.3. Maltogenic Amylase

Maltogenic amylase protein was expressed in *E. coli* BL21 (DE3) using pET102 Directional TOPO expression vector harboring the SK70 gene [29]. The bacteria were grown in 100 mL LB broth containing 50 µg/mL ampicillin. The culture was induced using IPTG at the final concentration of 0.02 mM when the OD_600nm_ reached to 0.6 and incubated for 12 h at 37 °C. Then, the culture was centrifuged at 10,000× *g* for 10 min at 4 °C. The pellet was resuspended in 50 mM phosphate buffer, pH 7.0 and sonicated for 2 min inclusive of the interval time. The intracellular crude Maltogenic amylase (free enzyme) was obtained by centrifugation of sonicated cells at 10,000× *g* for 15 min at 4 °C. The free enzyme was freeze dried at −45 °C until drying completely using Alpha 1-2 LD plus freeze dryer for 48 h to acquire the powder form of enzyme.

### 3.3. Enzyme Analysis

#### 3.3.1. Lipase

The residual activity of lipase was measured using Kwon and Rhee colorimetric assay method with slight modifications [3,31]. Olive oil was used as a substrate. The reaction mixture consisted of 2.5 mL of substrate emulsion (1:1 ratio of olive oil and 50 mM Glycine-NaOH, pH 9), 0.02 mL 20 mM CaCl_2_, 0.99 mL Glycine–NaOH buffer (pH 9.0) and 0.01 mL crude T1 lipase. This mixture was incubated in a water bath at 70 °C with 200 rpm shaking for 30 min. The reaction was stopped by adding 1 mL of 6 N HCl and then 5 mL of isooctane as an extraction solvent. Then, the mixture was vortexed for 30 s and left for 15 min. The upper layer (4 mL) was transferred into a test tube and 1 mL of cupric acetate pyridine reagent [Copper(II) acetate monohydrate (5%; pH 6.1), pH adjusted by adding pyridine] was added into the test tube and vortexed for 30 s. The mixture was left to settle for an hour. The free fatty acids dissolved in isooctane were determined by measuring the O.D. of the isooctane solution at 715 nm using Ultrospec 2100 Pro spectrophotometer (Amersham Bioscience, Uppsala, Sweden). One unit (U) of lipase activity is defined as the rate of 1 µmol of free fatty acid released per minute under standard assay conditions. All assays were conducted in triplicate.

#### 3.3.2. Rand Protease

Protease residual activity was determined by a method described by Abusham et al. [14]. Azocasein was used as a substrate. Reaction was initiated by adding 0.1 mL crude Rand protease into 1 mL azocasein solution (0.5% (*w*/*v*) azocasein dissolved in 0.1 M Tris-HCl, pH 7.0 and 2 mM CaCl_2_) and incubated in a water bath at 50 °C with 150 rpm shaking for 30 min. After 30 min, 1.1 mL of 10% (*w*/*v*) trichloroacetic acid (TCA) was added to terminate enzymatic reaction and stored at room temperature for 30 min. Then, 1.4 mL of the mixture was transferred into a micro-centrifuge tube and centrifuged at 13,000× *g* for 10 min. A volume of 0.7 mL supernatant was removed and mixed with an equal volume of 1 N NaOH. The absorbance was measured at 450 nm using Ultrospec 2100 Pro spectrophotometer (Amersham Bioscience, Uppsala, Sweden). One unit of protease activity is defined in the assay conditions, giving an increase of 0.001 absorbance unit at 450 nm per minute. As a control, the enzyme was added at the end of the incubation period. All assays were done in triplicate.

#### 3.3.3. Maltogenic Amylase

Amylase residual activity was assayed based on the Dinitrosalicylic acid (DNSA) method described by Winn-Deen et al. [32]. The reaction mixture consisted of 1.9 mL substrate (1% (*w*/*v*) cyclodextrin dissolved in 50 mM phosphate buffer, pH 7) and 0.1 mL crude Maltogenic amylase was incubated in the water bath at 55 °C for 30 min with 150 rpm shaking. After 30 min, the mixture was taken out from the water bath and 2 mL of DNSA reagent (A mixture of 1% (*w*/*v*) DNSA (3,5-dinitrosalicylic acid), 20 mL of 2 M NaOH and 30 g of Rochelle salt) was added and incubated in water bath at 100 °C for 10 min with 150 rpm shaking to stop the enzymatic reaction by denaturing the Maltogenic amylase. Then, it was cooled down at room temperature and the absorbance was measured at 540 nm using Ultrospec 2100 Pro spectrophotometer (Amersham Bioscience, Uppsala, Sweden). One unit (U) of amylase activity is defined as the amount of enzyme which releases 1 µmol of reducing end group sugar per minute under standard assay conditions. All assays were performed in triplicate.

### 3.4. Protein Assay

The protein contents of the crude enzymes were estimated using Bradford’s method as described by Bradford [33]. The protein content was calculated using absorbance reading obtained at 595 nm based on BSA standard curve. The protein concentration of powder form or encapsulated enzyme were measured before spray or freeze drying (in liquid form) to eliminate the interference of the gum arabic reading.

### 3.5. Encapsulation of Enzymes

Encapsulation efficiency is the ratio between enzyme activity of an immobilized enzyme or powdered free enzyme and liquid free enzyme:

Encapsulation efficiency = E/F × 100(1)

where E is activity of the encapsulated or powdered free enzyme and F is activity of the liquid free enzyme [19].

#### 3.5.1. T1 Lipase

The encapsulation was carried out as described by Rahman et al. [3]. The base matrix contained 6% (*w*/*v*) Gum arabic and 12% (*w*/*v*) maltodextrin, mixed with (3% *v*/*v*) crude enzyme, supernatant T1 lipase, in 50 mM glycine-NaOH buffer, pH 9.0. The mixture was then spray dried using BÜCHI mini spray dryer B-290 (St. Gallen, Switzerland) at inlet 160 °C, outlet 85 °C, feed rate of 25 mL/min and an air flow rate of 536 L/h. The spray dried powder was collected and enzyme residual activity was measured. Then, encapsulation efficiency was calculated.

#### 3.5.2. Rand Protease

The encapsulation of Rand protease was carried out with slight modifications of the method described by Mehrnoush et al. [19]. The combination of 3% (*w*/*v*) gum arabic and 6% (*w*/*v*) maltodextrin were used as a matrix with Rand protease as the crude enzyme. The mixture was incubated at 20 °C for 1 h at 150 rpm. Then, it was freeze dried at −45 °C for 48 h until it dried completely using Alpha 1-2 LD plus freeze dryer. The freeze-dried powder was collected and enzyme residual activity was checked, after which encapsulation efficiency was calculated.

#### 3.5.3. Maltogenic Amylase

The encapsulation of maltogenic amylase was carried out with slight modifications of the method that described by Mehrnoush et al. [19]. The matrix contained only 5.1% (*w*/*v*) gum arabic and crude maltogenic amylase in 50 mM phosphate (pH 7) added to the solution. The mixture was incubated at 20 °C for 1 h at 150 rpm. Then, it was freeze dried at −45 °C until drying completely using Alpha 1-2 LD plus freeze dryer for 48 h. The freeze dried powder was collected and enzyme residual activity was measured and encapsulation efficiency was calculated.

#### 3.5.4. Scanning Electron Microscope

The powder particles were viewed using a LEO 1455 variable pressure scanning electron microscope (VPSEM) located at the microscopy unit at the Institute of Bioscience, UPM, Selangor. The enzyme residual activity was checked using the method described above. The protein concentration was estimated using the same spectrophotometer at 280 nm, and a quartz cuvette was used.

### 3.6. Enzymes Compatibility Tests

The compatibility test of each enzyme with different components of detergent was tested by incubating the free enzyme in mixtures containing 0.2% (*v*/*v*) or (*w*/*v*) of detergent components such as surfactants (polyethylene glycol 300 (PEG300), glucopon 600 (G600), Tween 80 (T80) and sodium dodecyl sulfate (SDS)), bleaches (sodium percarbonate and sodium perborate), dispersing agents (sodium polyacrylate), builder (sodium citrate, sodium metasilicate and sodium silicate) and alkalinity agents (Phosphate, Tris-HCl, sodium citrate, sodium bicarbonate, a mixture of sodium carbonate-glycine, a mixture of sodium carbonate–sodium bicarbonate, and glycine–NaOH) for 30 min in the water bath at 60 °C [3]. Each enzyme was assayed for its residual activity at its optimum temperature; T1 lipase 70 °C, Rand protease 50 °C and Maltogenic amylase 55 °C. The control contained the enzyme without the tested compound which was incubated under similar conditions and considered as 100% activity.

### 3.7. Detergent Formulation

The formulated detergent was prepared by adding the components that had shown high stability for all three enzymes. All the enzymes compatibility test were done with 0.2% (*v*/*v*) or (*w*/*v*) of different components. Then, washing performance was determined to optimize the concentration of each selected component and find out the best concentration for detergent formulation (data is not shown). The optimized formulation contained 7% glucopon^®^ 600 CS UP (surfactant), 5% sodium polyacrylate (dispersing agent) and 3% sodium citrate (builder) in sodium carbonate–glycine (30:70) buffer, pH 9.25. The mixture was prepared and stored at 4 °C. For each dishwashing performance test, the same units of fresh free enzymes or immobilized enzymes were added and mixed thoroughly before use.

### 3.8. Hard Water Preparation

Hard water for the dishwashing test was artificially prepared by mixing magnesium sulfate and calcium chloride. The 5000 ppm stock was prepared first by adding 10 mM MgSO_4_·7H_2_O and 30 mM CaCl_2_·2H_2_O into 1 L deionized water [34]. For each test run, the stock was diluted and standardized to 350 ppm using a water hardness indicator (HI 96735 Hardness ISM, Hanna Instrument, Limena, Italy).

### 3.9. Dishwashing Test

Dishwashing test was done with slight modifications using Leenert’s Improved Detergency Tester (Japan) as described by Rahman et al. [3]. The soil used for washing was Skippy^®^ Peanut butter (20 g) immersed in 60 mL of acetone and stirred until it dissolved. The slides were weighed and they were dipped for 2 s into the soil bath and air-dried for 2 h. They were washed at 250 ± 10 rpm for 3 min, followed by 1 min of rinsing with same water hardness and temperature. The washed slides were air dried for 24 h and weighed again. The presence of soil removal was calculated with the following formula:

(2) $$\mathrm{Percentage}\;\mathrm{soil}\;\mathrm{removed}\;(\%)\;=\;\frac{W_{B}\;-\;W_{A}}{W_{B}}\times 100$$

where W_B_ is the weight of total soil before washing (mg) and W_A_ is the weight of total soil after washing (mg). All the washing and reading tests were done in triplicate to ensure the reproducibility.

#### 3.9.1. Efficiency of Detergent Formulation

The dishwashing tests were conducted in both soft and hard water at different water temperatures. This was intended to check the formulated detergents’ efficiency before the addition of enzymes. The soiled slides were weighed before washing. Then, slides were washed at 250 ± 10 rpm for 3 min using 1.5% (*w*/*v*) of formulated detergent without enzymes. The washing was followed by 1 min of rinsing with same water hardness and temperatures. The washed slides were air dried for 24 h and weighed.

#### 3.9.2. Determination of Individual Enzyme Concentration

The dishwashing tests were conducted in soft and hard water at a water temperature of 60 °C. The soiled slides were weighed before washing. Then, the slides were washed at 250 ± 10 rpm for 3 min using 1.5% (*w*/*v*) of formulated detergent containing different amount of enzyme, T1 lipase (1–7%), Rand protease (3–15%) and maltogenic amylase (1–2.5%). The washing was followed by 1 min of rinsing with same water hardness and temperature. The washed slides were air dried for 24 h and weighed. Each enzyme was characterized individually. Then, the amounts of enzymes which showed highest washing significant different were chosen based on post hoc test and incorporated into the formulated detergent.

#### 3.9.3. Comparison of Detergent with Free and Encapsulated Enzymes

Formulated detergents containing free and encapsulated enzymes were prepared and used. The experiments were done using 1.5% (*w*/*v*) of formulated detergent containing 3X of T1 lipase, 9Y of Rand protease and 1.5 Z of Maltogenic amylase. Washing was performed for free and encapsulated enzymes individually at temperatures of 21, 40, 50 and 60 °C in both soft and hard water. Soiled slides were washed at 250 ± 10 rpm for 3 min, followed by 1 min of rinsing with same water hardness and temperature. The washed slides were air dried for 24 h and weighed.

#### 3.9.4. Effect of Detergent Concentration on Removal of Soil

The dishwashing tests were conducted at a temperature of 50 °C in hard water. The experiments were done using different concentration of formulated detergent for washing with constant amount of enzymes. Soiled slides were washed at 250 ± 10 rpm for 3 min, followed by 1 min of rinsing with same water hardness and temperature. Then, the washed slides were air dried for 24 h and weighed.

#### 3.9.5. Effect of Washing Time on Removal of Soil

The dishwashing tests were conducted at a temperature of 50 °C in hard water. The formulated detergent, 1.5% (*w*/*v*), containing free and encapsulated enzymes were used for washing. The soiled slides were washed at 250 ± 10 rpm at different washing timings of 3, 5, 10, 15, 20, 25 and 30 min, followed by 1 min of rinsing with same water hardness and temperature. The washed slides were air dried for 24 h and weighed.

#### 3.9.6. Comparison of Formulated Detergent and Commercial

The washing performance of formulated detergent was compared with a commercial detergent, Finish^®^. The same amount of all detergents, 1.5% (*w*/*v*) was used for washing. Washings were done at different temperatures of 40, 50 and 60 °C in both soft and hard water. Soiled slides were washed at 250 ± 10 rpm for 3 min, followed by 1 min of rinsing with same water hardness and temperature. The washed slides were air dried for 24 h and weighed again.

### 3.10. Statistical Analysis

Statistical analyses were done using Tukey test (post hoc tests) at 0.05 level using SPSS Statistic 20.0 (SPSS Inc., Chicago, IL, USA).

## 4. Conclusions

Due to the concern towards the environment, the detergent industry has changed its approach to a more environmental friendly one, which includes biodegradable chemicals and enzymes. Hence, three Malaysian locally isolated enzymes, T1 lipase, Rand protease and Maltogenic amylase, were incorporated to formulate a new automatic dishwashing detergent with better efficiency in two forms of free and encapsulated enzymes. Compatible formulated detergent consisted of glucopon^®^ 600 CS UP (7%), sodium polyacrylate (5%) and sodium citrate (3%) in sodium carbonate-glycine (30:70) buffer, pH 9.25. The detergent containing encapsulated enzymes (Detergent B) works better than a detergent containing free enzymes (Detergent A) at the range of 50 to 60 °C, especially in hard water. In addition, Detergent B showed a higher percentage of soil removal at minute 30 compared to Detergent A. Hence, Detergent B has higher dishwashing efficiency in both soft and hard water and showed comparable efficiency to Finish^®^ in both soft and hard water at elevated temperatures.

## Figures and Tables

**Table 1:** Stability of enzymes in 0.2% (*v*/*v*) or (*w*/*v*) of various surfactants, bleach, dispersing agent, builders and alkalinity agents.

Parameter	Components	Types of Enzymes (Relative Activity (%))		
		T1 Lipase	Rand Protease	Maltogenic Amylase
**Control**	-	100	100	100
**surfactants**	PEG (non-ionic)	84.58 ± 0.04	94.96 ± 0.07	113.5 ± 0.01
	G600 (non-ionic)	108.57± 0.07	99.33 ± 0.05	101 ± 0.07
	Tween 80 (non-ionic)	98.8 ± 0.04	115.51 ± 0.06	93.39 ± 0.07
	SDS (anionic)	14 ± 0.03	10 ± 0.001	1.08 ± 0.08
**Bleach**	Sodium percarbonate	5.44 ± 0.06	5.2 ± 0.05	21.81 ± 0.60
	Sodium perborate	6.40 ± 0.07	24.32 ± 0.19	1.2 ± 0.50
**Dispersing agent**	Sodium polyacrylate	54 ± 0.18	48 ± 0.13	71.9 ± 0.03
**Builders**	Sodium citrate	48 ± 0.03	44.74 ± 0.04	96 ± 0.05
	Sodium metasillicate	7.55 ± 0.06	16.65 ± 0.02	0.3 ± 0.05
	Sodium silicate	20.8 ± 0.30	16.43 ± 0.04	0.68 ± 0.04
**Control**	Glycine-NaOH, pH 9.0	100	100	100
**Alkalinity agents**	Phosphate, pH 7.0	88.4 ± 0.09	100.3 ± 0.01	125 ± 0.02
	Tris-HCl, pH 7.0	42 ± 0.04	106 ± 0.10	64.4 ± 0.21
	Sodium citrate, pH 8.3	48 ± 0.03	54.74 ± 0.04	96 ± 0.05
	Sodium bicarbonate (SB), pH 8.6	80.7 ± 0.04	83.3 ± 0.27	129 ± 0.06
	Sodium carbonate (SC): glycine (30:70), pH 9.25	120 ± 0.17	92 ± 0.01	119.1 ± 0.2
	SC:SB (30:70), pH 9.5	5 ± 0.05	67.9 ± 0.02	70 ± 0.08

Note: Data are means ± standard deviation of three determinations.

**Table 2:** Enzymatic activity performance of encapsulated enzymes.

Enzymes		Encapsulated Enzyme	Powdered Free Enzyme	Control (Liquid Free Enzyme)
**T1 lipase**	Total activity (U)	1048.3	420	1098
	Activity retained (%)	95.5	38.25	100
**Rand protease**	Total activity (U)	10289	5032.5	11250
	Activity retained (%)	91.4	44.73	100
**Maltogenic amylase**	Total activity (U)	744.4	31.26	990
	Activity retained (%)	75.2	3.2	100

**Table 3:** Effect of detergent concentration on soil removal.

Detergent Concentration (%)	Percentage of Soil Removal	
	**Detergent A**	**Detergent B**
**0**	8.3 ± 1.2 ^p1^	8.3 ± 1.2 ^q1^
**1.5**	41.4 ± 1.8 ^p2^	49.6 ± 0.3 ^q2^
**2**	44.0 ± 1.7 ^p2^	51.0 ± 1.4 ^q2^
**2.5**	44.6 ± 1.8 ^p2^	51.3 ± 1.4 ^q2^

Note: Superscripts p1 and p2 (test using detergent A) and q1 and q2 (test using detergent B) indicated groups that showed a significant difference between the groups when different detergent concentrations were used. All superscripts were obtained using post-hoc tests as shown in Table S5. Data are means ± standard deviation of three determinations.

## Supplementary Materials

Supplementary materials are available online. The statistical analysis of one-way ANOVA and Post Hoc test and Detergent performance profiles using the 3 enzymes (lipase, protease and amylase) in soft and hard water are available online.
