# Supplementary material for: Improving the Efficiency of New Automatic Dishwashing Detergent Formulation by Addition of Thermostable Lipase, Protease and Amylase
Source: Molecules. 2017 Sep 19;22(9):1577. doi: 10.3390/molecules22091577 (PMC6151835; doi:10.3390/molecules22091577)
Supplement: Supplementary file 1 [file molecules-22-01577-s001.pdf]

Supplementary information

Article

Improving the efficiency of new automatic dishwashing detergent formulation by addition of thermostable lipase, protease and amylase

Ashwini Naganthran [1,2], Malihe Masomian [1, 2], Mohd Shukuri Mohamad Ali [1, 3], Hisham Mohd Nooh[1,4] and Raja Noor Zaliha Raja Abd. Rahman [1, 2]

[1] Enzyme and Microbial Technology Research Center, Universiti Putra Malaysia, Serdang, Selangor, 43400, Malaysia
[2] Department of Microbiology, Faculty of Biotechnology and Biomolecular Science, Universiti Putra Malaysia, Serdang, Selangor, 43400, Malaysia
[3] Department of Biochemistry, Faculty of Biotechnology and Biomolecular Science, Universiti Putra Malaysia, Serdang, Selangor, 43400, Malaysia
[4] Laboratory of Molecular Biomedicine, Institute of Bioscience, Universiti Putra Malaysia, Serdang, Selangor, 43400, Malaysia
* Correspondence: rnzaliha@upm.edu.my (R.N.Z.R.A. Rahman); Tel.: +60 3 8947 1416

Academic Editor: name

Table S1: Statistical analysis of one-way ANOVA and Post Hoc test on efficiency of formulated detergent.

One- way ANOVA for data on Figure 1A

ANOVA

				Sum of Squares	df	Mean Square	F	Sig.
Without detergent	Between Groups	(Combined)		1128.047	3	376.016	99.227	.000
		Linear Term	Contrast	983.733	1	983.733	259.598	.000
			Deviation	144.314	2	72.157	19.042	.009
	Within Groups			15.158	4	3.789		
	Total			1143.205	7			
With detergent	Between Groups	(Combined)		8090.910	3	2696.970	3739.559	.000
		Linear Term	Contrast	6204.158	1	6204.158	8602.549	.000
			Deviation	1886.752	2	943.376	1308.064	.000
	Within Groups			2.885	4	.721		
	Total			8093.795	7			

Post Hoc test for Figure 1A

Without detergent

Tukey HSD

temperature	N	Subset for alpha = 0.05	
		1	2
21	2	6.0000	
40	2		30.5200
50	2		33.4000
60	2		35.4500
Sig.		1.000	.193

Means for groups in homogeneous subsets are displayed.

a. Uses Harmonic Mean Sample Size = 2.000.

With detergent

Tukey HSD

temperature	N	Subset for alpha = 0.05	
		1	2
21	2	10.0600	
40	2		83.2000
50	2		83.3000
60	2		84.0000
Sig.		1.000	.787

Means for groups in homogeneous subsets are displayed.

a. Uses Harmonic Mean Sample Size = 2.000.

One- way ANOVA for data on Figure 1B

ANOVA

				Sum of Squares	df	Mean Square	F	Sig.
Without detergent	Between Groups	(Combined)		64.564	3	21.521	344.340	.000
		Linear Term	Contrast	63.621	1	63.621	1017.936	.000
			Deviation	.943	2	.471	7.542	.044
	Within Groups			.250	4	.063		
	Total			64.814	7			
With detergent	Between Groups	(Combined)		515.638	3	171.879	85.321	.000
		Linear Term	Contrast	474.656	1	474.656	235.620	.000
			Deviation	40.982	2	20.491	10.172	.027
	Within Groups			8.058	4	2.015		
	Total			523.696	7			

Post Hoc test for Figure 1B

Without detergent

Tukey HSD

temperature	N	Subset for alpha = 0.05 1	2	3	4
21.00	2	2.0000			
40.00	2		5.6000		
50.00	2			8.3000	
60.00	2				9.3500
Sig.		1.000	1.000	1.000	1.000

Means for groups in homogeneous subsets are displayed.

a. Uses Harmonic Mean Sample Size = 2.000.

With detergent

Tukey HSD

temperature	N	Subset for alpha = 0.05 1	2
21.00	2	3.0000	
40.00	2		18.7900
50.00	2		20.1500
60.00	2		24.0500
Sig.		1.000	.067

Means for groups in homogeneous subsets are displayed.

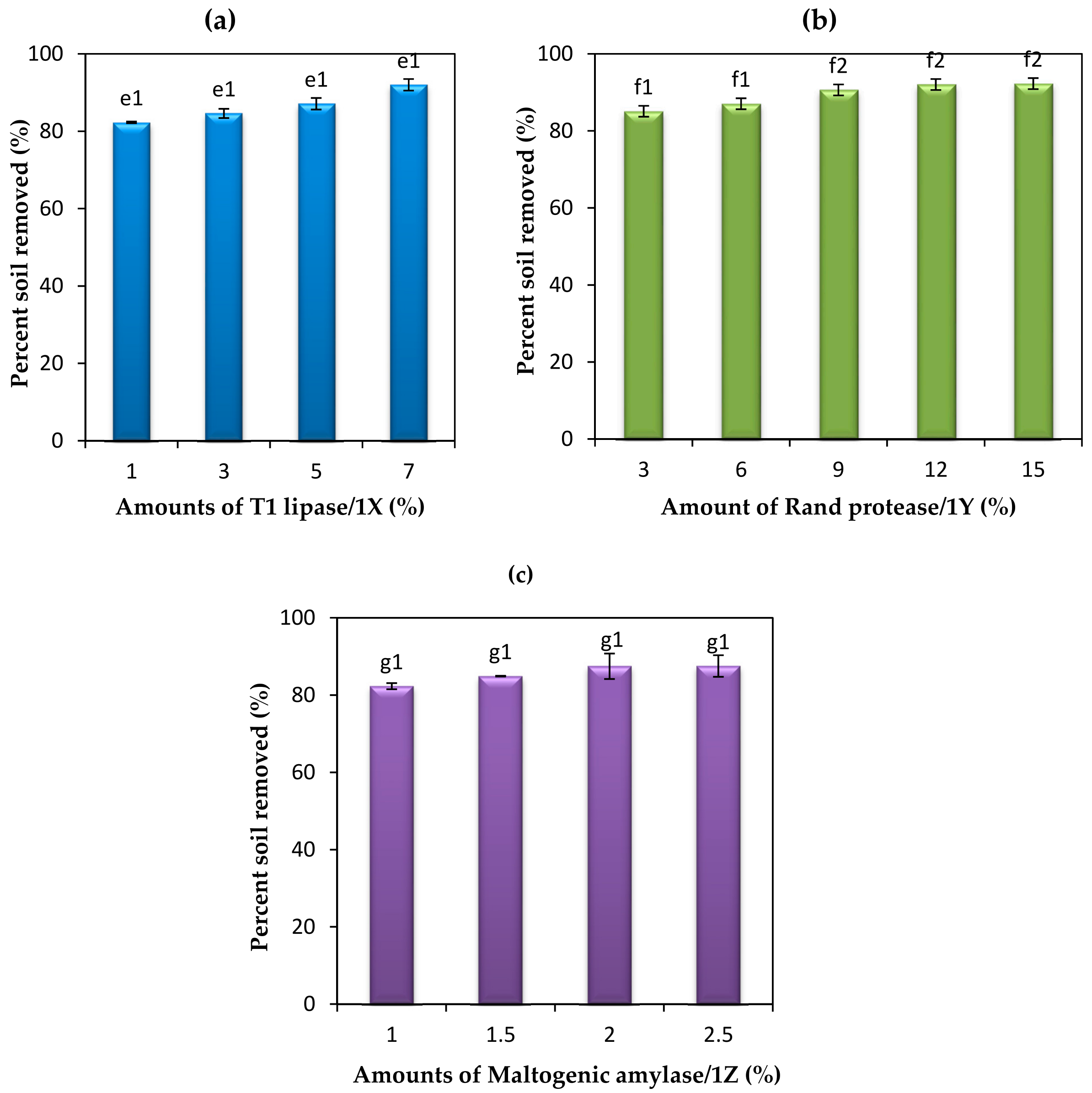

Figure S1. Detergent performance profiles using all 3 enzymes (lipase, protease and amylase). The dishwashing test was carried out in soft water (0 ppm $CaCO_3$) at 60°C. The detergent was contained 7 % surfactant, 3 % builders, 5 % dispersing agent in glycine-carbonate (pH 9.25) buffer and the selected amount of enzymes. The (a) T1 lipase, (b) Rand protease and (c) Maltogenic amylase concentration were multiple of 1X, 1Y and 1Z, respectively.

Note: Superscript f2 indicated groups that showed a significant difference between the groups when different enzyme concentration was added. All superscripts were obtained using post-hoc tests as shown in Table S2. Data are means ± standard deviation of three determinations and indicated as error bars.

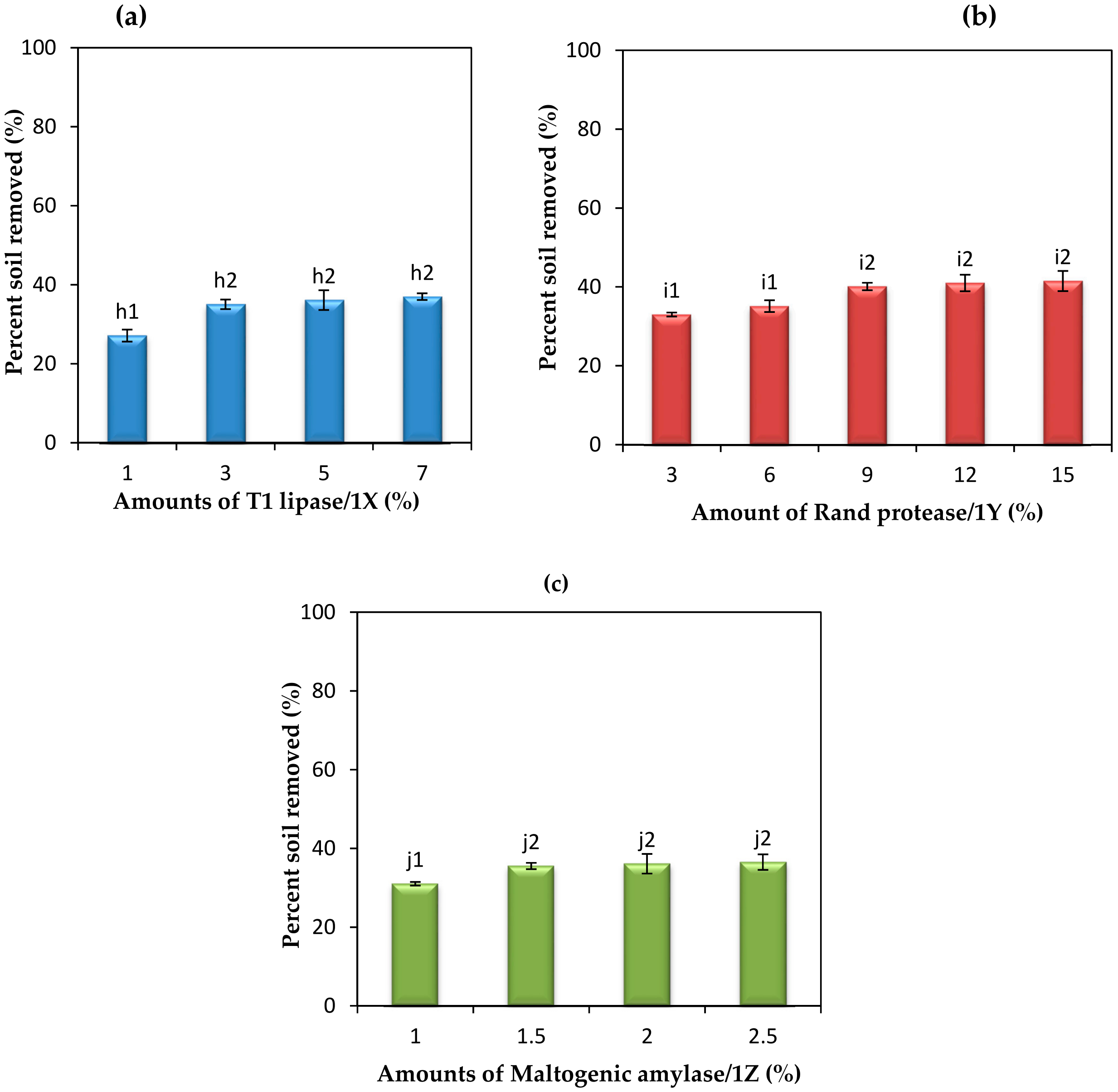

Figure S2. Detergent performance profiles using all 3 enzymes (lipase, protease and amylase). The dishwashing test was carried out in hard water (350 ppm $CaCO_3$) at 60°C. The detergent was contained 7 % surfactant, 3 % builders, 5 % dispersing agent in glycine-carbonate (pH 9.25) buffer and the selected amount of enzymes. The (a) T1 lipase, (b) Rand protease and (c) Maltogenic amylase concentration were multiple of 1X, 1Y and 1Z, respectively.

Note: Superscript h2, i2 and j2 indicated groups that showed a significant difference between the groups when different enzyme concentrations were added. All superscripts were obtained using post-hoc tests as shown in Table S3. Data are means ± standard deviation of three determinations and indicate as error bars.

Table S2: Statistical analysis of one-way ANOVA and Post Hoc test on detergent performance profiles using all 3 enzymes (lipase, protease and amylase) in soft water.

One- way ANOVA for data on Figure S1A

ANOVA

Soil removal (T1 lipase addition)

	Sum of Squares	df	Mean Square	F	Sig.
Between Groups	103.161	3	34.387	3.926	.110
Within Groups	35.036	4	8.759		
Total	138.197	7			

Post Hoc Tests for Figure S1A

Multiple Comparisons

Dependent Variable:

Tukey HSD

(I) percentage	(J) percentage	Mean Difference (I-J)	Std. Error	Sig.	95% Confidence Interval	
					Lower Bound	Upper Bound
1.00	3.00	-2.26665	2.95954	.866	-14.3145	9.7812
	5.00	-4.76665	2.95954	.465	-16.8145	7.2812
	7.00	-9.66665	2.95954	.098	-21.7145	2.3812
3.00	.00	2.26665	2.95954	.866	-9.7812	14.3145
	5.00	-2.50000	2.95954	.832	-14.5479	9.5479
	7.00	-7.40000	2.95954	.199	-19.4479	4.6479
5.00	.00	4.76665	2.95954	.465	-7.2812	16.8145
	3.00	2.50000	2.95954	.832	-9.5479	14.5479
	7.00	-4.90000	2.95954	.446	-16.9479	7.1479
7.00	.00	9.66665	2.95954	.098	-2.3812	21.7145
	3.00	7.40000	2.95954	.199	-4.6479	19.4479
	5.00	4.90000	2.95954	.446	-7.1479	16.9479

One- way ANOVA for data on Figure S1B

ANOVA

Soil removal (Rand protease addition)

	Sum of Squares	df	Mean Square	F	Sig.
Between Groups	172.248	5	34.450	8.005	.012
Within Groups	25.820	6	4.303		
Total	198.068	11			

Post Hoc Tests for Figure S1B

Multiple Comparisons

Dependent Variable: soil
Tukey HSD

(I) percentage	(J) percentage	Mean Difference (I-J)	Std. Error	Sig.	95% Confidence Interval	
					Lower Bound	Upper Bound
3.00	.00	2.74667	2.07444	.766	-5.5093	11.0026
	6.00	-.92000	2.07444	.997	-9.1759	7.3359
	9.00	-5.55500	2.07444	.210	-13.8109	2.7009
	12.00	-6.95333	2.07444	.099	-15.2093	1.3026
	15.00	-7.19750	2.07444	.087	-15.4534	1.0584
6.00	.00	3.66667	2.07444	.542	-4.5893	11.9226
	3.00	.92000	2.07444	.997	-7.3359	9.1759
	9.00	-4.63500	2.07444	.341	-12.8909	3.6209
	12.00	-6.03333	2.07444	.162	-14.2893	2.2226
	15.00	-6.27750	2.07444	.142	-14.5334	1.9784
9.00	.00	8.30167*	2.07444	.049	.0457	16.5576
	3.00	5.55500	2.07444	.210	-2.7009	13.8109
	6.00	4.63500	2.07444	.341	-3.6209	12.8909
	12.00	-1.39833	2.07444	.979	-9.6543	6.8576
	15.00	-1.64250	2.07444	.959	-9.8984	6.6134
12.00	.00	9.70000*	2.07444	.025	1.4441	17.9559
	3.00	6.95333	2.07444	.099	-1.3026	15.2093
	6.00	6.03333	2.07444	.162	-2.2226	14.2893
	9.00	1.39833	2.07444	.979	-6.8576	9.6543
	15.00	-.24417	2.07444	1.000	-8.5001	8.0118
15.00	.00	9.94417*	2.07444	.022	1.6882	18.2001
	3.00	7.19750	2.07444	.087	-1.0584	15.4534
	6.00	6.27750	2.07444	.142	-1.9784	14.5334
	9.00	1.64250	2.07444	.959	-6.6134	9.8984
	12.00	.24417	2.07444	1.000	-8.0118	8.5001

*. The mean difference is significant at the 0.05 level.

One- way ANOVA for data on Figure S1C

ANOVA

Soil removal (maltogenic amylase addition)

	Sum of Squares	df	Mean Square	F	Sig.
Between Groups	36.523	3	12.174	1.414	.362
Within Groups	34.429	4	8.607		
Total	70.951	7			

Post Hoc Tests for Figure S1C

Multiple Comparisons

Tukey HSD

(I) percentage	(J) percentage	Mean Difference (I-J)	Std. Error	Sig.	95% Confidence Interval	
					Lower Bound	Upper Bound
	1.50	-2.57915	2.93381	.816	-14.5223	9.3640
1.00	2.00	-5.14165	2.93381	.407	-17.0848	6.8015
	2.50	-5.16665	2.93381	.404	-17.1098	6.7765
	.00	2.57915	2.93381	.816	-9.3640	14.5223
1.50	2.00	-2.56250	2.93381	.819	-14.5056	9.3806
	2.50	-2.58750	2.93381	.815	-14.5306	9.3556
	.00	5.14165	2.93381	.407	-6.8015	17.0848
2.00	1.50	2.56250	2.93381	.819	-9.3806	14.5056
	2.50	-.02500	2.93381	1.000	-11.9681	11.9181
	.00	5.16665	2.93381	.404	-6.7765	17.1098
2.50	1.50	2.58750	2.93381	.815	-9.3556	14.5306
	2.00	.02500	2.93381	1.000	-11.9181	11.9681

Table S3: Statistical analysis of one-way ANOVA and Post Hoc test on detergent performance profiles using all 3 enzymes (lipase, protease and amylase) in hard water.

One- way ANOVA for data on Figure S2A

ANOVA

removal

	Sum of Squares	df	Mean Square	F	Sig.
Between Groups	183.849	3	61.283	22.846	.000
Within Groups	21.460	8	2.682		
Total	205.309	11			

Post Hoc Tests for Figure S2A

Multiple Comparisons

Dependent Variable: removal

Tukey HSD

(I) amount	(J) amount	Mean Difference (I-J)	Std. Error	Sig.	95% Confidence Interval	
					Lower Bound	Upper Bound
1.00	3.00	-7.90000000*	1.33728581	.002	-12.1824599	-3.6175401
	5.00	-8.96666667*	1.33728581	.001	-13.2491266	-4.6842068
	7.00	-9.83333333*	1.33728581	.000	-14.1157932	-5.5508734
3.00	1.00	7.90000000*	1.33728581	.002	3.6175401	12.1824599
	5.00	-1.06666667	1.33728581	.854	-5.3491266	3.2157932
	7.00	-1.93333333	1.33728581	.508	-6.2157932	2.3491266
5.00	1.00	8.96666667*	1.33728581	.001	4.6842068	13.2491266
	3.00	1.06666667	1.33728581	.854	-3.2157932	5.3491266
	7.00	-.86666667	1.33728581	.913	-5.1491266	3.4157932
7.00	1.00	9.83333333*	1.33728581	.000	5.5508734	14.1157932
	3.00	1.93333333	1.33728581	.508	-2.3491266	6.2157932
	5.00	.86666667	1.33728581	.913	-3.4157932	5.1491266

*. The mean difference is significant at the 0.05 level.

Homogeneous Subsets

removal

Tukey HSD

amount	N	Subset for alpha = 0.05	
		1	2
1.00	3	27.1333333	
3.00	3		35.0333333
5.00	3		36.1000000
7.00	3		36.9666667
Sig.		1.000	.508

Means for groups in homogeneous subsets are displayed.

a. Uses Harmonic Mean Sample Size = 3.000.

One- way ANOVA for data on Figure S2B

ANOVA

removal

	Sum of Squares	df	Mean Square	F	Sig.
Between Groups	183.849	3	61.283	22.846	.000
Within Groups	21.460	8	2.682		
Total	205.309	11			

Post Hoc Tests for Figure S2B

Multiple Comparisons

Dependent Variable: removal

Tukey HSD

(I) amount	(J) amount	Mean Difference (I-J)	Std. Error	Sig.	95% Confidence Interval	
					Lower Bound	Upper Bound
1.00	3.00	-7.90000000*	1.33728581	.002	-12.1824599	-3.6175401
	5.00	-8.96666667*	1.33728581	.001	-13.2491266	-4.6842068
	7.00	-9.83333333*	1.33728581	.000	-14.1157932	-5.5508734
3.00	1.00	7.90000000*	1.33728581	.002	3.6175401	12.1824599
	5.00	-1.06666667	1.33728581	.854	-5.3491266	3.2157932
	7.00	-1.93333333	1.33728581	.508	-6.2157932	2.3491266
5.00	1.00	8.96666667*	1.33728581	.001	4.6842068	13.2491266
	3.00	1.06666667	1.33728581	.854	-3.2157932	5.3491266
	7.00	-.86666667	1.33728581	.913	-5.1491266	3.4157932
7.00	1.00	9.83333333*	1.33728581	.000	5.5508734	14.1157932
	3.00	1.93333333	1.33728581	.508	-2.3491266	6.2157932
	5.00	.86666667	1.33728581	.913	-3.4157932	5.1491266

*. The mean difference is significant at the 0.05 level.

Homogeneous Subsets

removal

Tukey HSD

amount	N	Subset for alpha = 0.05	
		1	2
1.00	3	27.1333333	
3.00	3		35.0333333
5.00	3		36.1000000
7.00	3		36.9666667
Sig.		1.000	.508

Means for groups in homogeneous subsets are displayed.

a. Uses Harmonic Mean Sample Size = 3.000.

One- way ANOVA for data on Figure S2C

ANOVA

removal

	Sum of Squares	df	Mean Square	F	Sig.
Between Groups	57.772	3	19.257	7.135	.012
Within Groups	21.593	8	2.699		
Total	79.365	11			

Post Hoc Tests for Figure S2C

Multiple Comparisons

Dependent Variable: removal

Tukey HSD

(I) amount	(J) amount	Mean Difference (I-J)	Std. Error	Sig.	95% Confidence Interval	
					Lower Bound	Upper Bound
1	1.50000	-4.50000000*	1.34142544	.040	-8.7957165	-.2042835
	2.00000	-5.07000000*	1.34142544	.022	-9.3657165	-.7742835
	2.50000	-5.45000000*	1.34142544	.015	-9.7457165	-1.1542835
1.50000	1	4.50000000*	1.34142544	.040	.2042835	8.7957165
	2.00000	-.57000000	1.34142544	.973	-4.8657165	3.7257165
	2.50000	-.95000000	1.34142544	.891	-5.2457165	3.3457165
2.00000	1	5.07000000*	1.34142544	.022	.7742835	9.3657165
	1.50000	.57000000	1.34142544	.973	-3.7257165	4.8657165
	2.50000	-.38000000	1.34142544	.991	-4.6757165	3.9157165
2.50000	1	5.45000000*	1.34142544	.015	1.1542835	9.7457165
	1.50000	.95000000	1.34142544	.891	-3.3457165	5.2457165
	2.00000	.38000000	1.34142544	.991	-3.9157165	4.6757165

*. The mean difference is significant at the 0.05 level.

Homogeneous Subsets

removal

Tukey HSD

amount	N	Subset for alpha = 0.05	
		1	2
1	3	31.0333333	
1.50000	3		35.5333333
2.00000	3		36.1033333
2.50000	3		36.4833333
Sig.		1.000	.891

Means for groups in homogeneous subsets are displayed.

a. Uses Harmonic Mean Sample Size = 3.000.

Table S4: Statistical analysis of one-way ANOVA and Post Hoc test on dishwashing performances of the formulated detergent with free and encapsulated enzymes.

One-way ANOVA on data for Figure 3A

ANOVA

D + without enzymes (soft water)

			Sum of Squares	df	Mean Square	F	Sig.
Between Groups	(Combined)		8090.910	3	2696.970	3739.559	.000
	Linear Term	Contrast	6204.158	1	6204.158	8602.549	.000
		Deviation	1886.752	2	943.376	1308.064	.000
Within Groups			2.885	4	.721		
Total			8093.795	7			

D + free enzymes (soft water)

Between Groups	(Combined)		9730.885	3	3243.628	737.119	.000
	Linear Term	Contrast	7791.137	1	7791.137	1770.547	.000
		Deviation	1939.748	2	969.874	220.405	.000
Within Groups			17.602	4	4.400		
Total			9748.486	7			

D + encapsulated enzymes (soft water)

Between Groups	(Combined)		9864.935	3	3288.312	1756.108	.000
	Linear Term	Contrast	8688.701	1	8688.701	4640.161	.000
		Deviation	1176.234	2	588.117	314.081	.000
Within Groups			7.490	4	1.872		
Total			9872.425	7			

Post Hoc test for Figure 3A

detergent + without enzymes (soft water)

Tukey HSD

		Subset for alpha = 0.05	
temperature	N	1	2
21	2	10.06	
40	2		83.20
50	2		83.30
60	2		84.00
Sig.		1.000	.787

Means for groups in homogeneous subsets are displayed.

detergent + free ezymes

Tukey HSD

temperature	N	Subset for alpha = 0.05	
		1	2
21	2	10.00	
40	2		88.1150
50	2		90.1600
60	2		93.0500
Sig.		1.000	.229

Means for groups in homogeneous subsets are displayed.

a. Uses Harmonic Mean Sample Size = 2.000.

detergent + encapsulated enzymes

Tukey HSD

temperature	N	Subset for alpha = 0.05		
		1	2	3
21	2	11.0000		
40	2		81.0500	
50	2			94.2000
60	2			97.3300
Sig.		1.000	1.000	.244

Means for groups in homogeneous subsets are displayed.

a. Uses Harmonic Mean Sample Size = 2.000.

One-way ANOVA on data for Figure 3A

ANOVA

(21 °c, soft water)			Sum of Squares	df	Mean Square	F	Sig.
Between Groups	(Combined)		1.258	2	.629	.495	.652
	Linear Term	Contrast	.884	1	.884	.695	.466
		Deviation	.375	1	.375	.295	.625
Within Groups			3.815	3	1.272		
Total			5.073	5			
**(40 °c, soft water)**							
Between Groups	(Combined)		52.463	2	26.231	14.483	.029
	Linear Term	Contrast	4.623	1	4.623	2.552	.208
		Deviation	47.840	1	47.840	26.414	.014
Within Groups			5.433	3	1.811		
Total			57.896	5			
**50 °c, soft water**							
Between Groups	(Combined)		121.461	2	60.730	67.901	.003
	Linear Term	Contrast	118.810	1	118.810	132.838	.001
		Deviation	2.651	1	2.651	2.964	.184
Within Groups			2.683	3	.894		
Total			124.144	5			
**60 °c, soft water**							
Between Groups	(Combined)		185.273	2	92.637	17.321	.023
	Linear Term	Contrast	177.689	1	177.689	33.223	.010
		Deviation	7.584	1	7.584	1.418	.319
Within Groups			16.045	3	5.348		

Post Hoc test for Figure 3A

Multiple Comparisons (21 °c, soft water)

Dependent Variable: removal
Tukey HSD

(I) type		Mean Difference (I-J)	Std. Error	Sig.	95% Confidence Interval Lower Bound	95% Confidence Interval Upper Bound
D	free	.06000	1.12765	.998	-4.6521	4.7721
	encap	-.94000	1.12765	.711	-5.6521	3.7721
free	D	-.06000	1.12765	.998	-4.7721	4.6521
	encap	-1.00000	1.12765	.684	-5.7121	3.7121
encap	D	.94000	1.12765	.711	-3.7721	5.6521
	free	1.00000	1.12765	.684	-3.7121	5.7121

Multiple Comparisons (40 °c, soft water)

Dependent Variable: removal
Tukey HSD

(I) type		Mean Difference (I-J)	Std. Error	Sig.	95% Confidence Interval	
					Lower Bound	Upper Bound
D	free	-4.91500	1.34579	.070	-10.5387	.7087
	encap	2.15000	1.34579	.372	-3.4737	7.7737
free	D	4.91500	1.34579	.070	-.7087	10.5387
	encap	7.06500*	1.34579	.027	1.4413	12.6887
encap	D	-2.15000	1.34579	.372	-7.7737	3.4737
	free	-7.06500*	1.34579	.027	-12.6887	-1.4413

Multiple Comparisons 50 °c, soft water

Dependent Variable: removal
Tukey HSD

(I) type		Mean Difference (I-J)	Std. Error	Sig.	95% Confidence Interval	
					Lower Bound	Upper Bound
D	free	-6.86000*	.94573	.011	-10.8119	-2.9081
	encap	-10.90000*	.94573	.003	-14.8519	-6.9481
free	D	6.86000*	.94573	.011	2.9081	10.8119
	encap	-4.04000*	.94573	.047	-7.9919	-.0881
encap	D	10.90000*	.94573	.003	6.9481	14.8519
	free	4.04000*	.94573	.047	.0881	7.9919

*. The mean difference is significant at the 0.05 level.

Multiple Comparisons (60 °c, soft water)

Dependent Variable: removal
Tukey HSD

(I) type		Mean Difference (I-J)	Std. Error	Sig.	95% Confidence Interval	
					Lower Bound	Upper Bound
D	free	-9.05000	2.31265	.059	-18.7139	.6139
	encap	-13.33000*	2.31265	.021	-22.9939	-3.6661
free	D	9.05000	2.31265	.059	-.6139	18.7139
	encap	-4.28000	2.31265	.296	-13.9439	5.3839
encap	D	13.33000*	2.31265	.021	3.6661	22.9939
	free	4.28000	2.31265	.296	-5.3839	13.9439

*. The mean difference is significant at the 0.05 level.

One-way ANOVA on data of Figure 3B

ANOVA

21°C , hard water			Sum of Squares	df	Mean Square	F	Sig.
Between Groups	(Combined)		.002	2	.001	.001	.999
	Linear Term	Contrast	0.000	1	0.000	0.000	1.000
		Deviation	.002	1	.002	.002	.964
Within Groups			2.074	3	.691		
Total			2.076	5			

40°C , hard water			Sum of Squares	df	Mean Square	F	Sig.
Between Groups	(Combined)		815.822	2	407.911	52.448	.005
	Linear Term	Contrast	762.312	1	762.312	98.016	.002
		Deviation	53.510	1	53.510	6.880	.079
Within Groups			23.332	3	7.777		
Total			839.154	5			

50°C , hard water			Sum of Squares	df	Mean Square	F	Sig.
Between Groups	(Combined)		923.534	2	461.767	17.583	.022
	Linear Term	Contrast	867.214	1	867.214	33.021	.010
		Deviation	56.320	1	56.320	2.145	.239
Within Groups			78.787	3	26.262		
Total			1002.321	5			

60°C , hard water			Sum of Squares	df	Mean Square	F	Sig.
Between Groups	(Combined)		713.370	2	356.685	103.479	.002
	Linear Term	Contrast	673.403	1	673.403	195.363	.001
		Deviation	39.968	1	39.968	11.595	.042
Within Groups			10.341	3	3.447		
Total			723.711	5			

Post Hoc test for Figure 3B

With detergent

Tukey HSD

temperature	N	Subset for alpha = 0.05	
		1	2
21.00	2	3.0000	
40.00	2		18.7900
50.00	2		20.1500
60.00	2		24.0500
Sig.		1.000	.067

Means for groups in homogeneous subsets are displayed.

Detergent + Free enzymes (hard water)

Tukey HSD

temperature	N	Subset for alpha = 0.05		
		1	2	3
21	2	3.0350		
40	2		26.2600	
50	2			41.3750
60	2			42.5000
Sig.		1.000	1.000	.831

Means for groups in homogeneous subsets are displayed.

a. Uses Harmonic Mean Sample Size = 2.000.

Detergent + Encapsulated enzymes (hard water)

Tukey HSD

temperature	N	Subset for alpha = 0.05	
		1	2
21	2	3.0000	
40	2		46.4000
50	2		49.6000
60	2		50.0000
Sig.		1.000	.081

Means for groups in homogeneous subsets are displayed.

a. Uses Harmonic Mean Sample Size = 2.000.

Post Hoc test for Figure 3B

Multiple Comparisons (40°C , hard water)

Dependent Variable: removal
Tukey HSD

(I) type		Mean Difference (I-J)	Std. Error	Sig.	95% Confidence Interval Lower Bound	Upper Bound
detergent	free	-7.47000	2.78880	.145	-19.1236	4.1836
	encap	-27.61000*	2.78880	.004	-39.2636	-15.9564
free	detergent	7.47000	2.78880	.145	-4.1836	19.1236
	encap	-20.14000*	2.78880	.011	-31.7936	-8.4864
encap	detergent	27.61000*	2.78880	.004	15.9564	39.2636
	free	20.14000*	2.78880	.011	8.4864	31.7936

*. The mean difference is significant at the 0.05 level.

Multiple Comparisons (50°C , hard water)

Dependent Variable: removal
Tukey HSD

(I) type		Mean Difference (I-J)	Std. Error	Sig.	95% Confidence Interval Lower Bound	Upper Bound
detergent only	free	-21.22350	5.12466	.051	-42.6380	.1910
	encap	-29.44850*	5.12466	.021	-50.8630	-8.0340
free	detergent	21.22350	5.12466	.051	-.1910	42.6380
	encap	-8.22500	5.12466	.369	-29.6395	13.1895
encap	detergent	29.44850*	5.12466	.021	8.0340	50.8630
	free	8.22500	5.12466	.369	-13.1895	29.6395

*. The mean difference is significant at the 0.05 level.

Multiple Comparisons (60°C , hard water)

Dependent Variable: removal
Tukey HSD

(I) type		Mean Difference (I-J)	Std. Error	Sig.	95% Confidence Interval Lower Bound	Upper Bound
detergent	free	-18.45000*	1.85659	.004	-26.2082	-10.6918
	encap	-25.95000*	1.85659	.002	-33.7082	-18.1918
free	detergent	18.45000*	1.85659	.004	10.6918	26.2082
	encap	-7.50000	1.85659	.055	-15.2582	.2582
encap	detergent	25.95000*	1.85659	.002	18.1918	33.7082
	free	7.50000	1.85659	.055	-.2582	15.2582

*. The mean difference is significant at the 0.05 level.

Table S5: Statistical analysis of Post Hoc test on effect of detergent concentration on soil removal.

Post hoc test within groups for Table 3

d + encapsulated enzymes

Tukey HSD

detergent	N	Subset for alpha = 0.05	
		1	2
.00	2	8.3000	
1.50	2		49.6000
1.5	2		51.0000
2.50	2		51.2500
Sig.		1.000	.626

Means for groups in homogeneous subsets are displayed.

a. Uses Harmonic Mean Sample Size = 2.000.

d + free enzymes

Tukey HSD

detergent	N	Subset for alpha = 0.05	
		1	2
.00	2	8.3000	
1.50	2		41.3750
2	2		44.0500
2.50	2		44.6000
Sig.		1.000	.902

Means for groups in homogeneous subsets are displayed.

a. Uses Harmonic Mean Sample Size = 2.000.

Table S6: Statistical analysis of one-way ANOVA and Post Hoc test on effect of washing time on soil removal using the formulated detergent A (contained free enzymes) and B (contained encapsulated enzymes) in hard water (350 ppm $CaCO_3$) at 50 °C.

One- way ANOVA on data within group for Figure 4

ANOVA

				Sum of Squares	df	Mean Square	F	Sig.
free	Between Groups	(Combined)		5077.050	6	846.175	74.062	.000
		Linear Term	Contrast	4094.158	1	4094.158	358.344	.000
			Deviation	982.892	5	196.578	17.206	.001
	Within Groups			79.977	7	11.425		
	Total			5157.027	13			
encap	Between Groups	(Combined)		3912.960	6	652.160	183.929	.000
		Linear Term	Contrast	3702.736	1	3702.736	1044.285	.000
			Deviation	210.224	5	42.045	11.858	.003
	Within Groups			24.820	7	3.546		
	Total			3937.780	13			

Post hoc test within group for Figure 4

free

Tukey HSD

		Subset for alpha = 0.05			
mins	N	1	2	3	4
3	2	41.3750			
5	2	45.5850			
10	2		63.3500		
15	2		76.5000	76.5000	
30	2			84.0500	84.0500
25	2			88.0000	88.0000
20	2				92.7050
Sig.		.857	.054	.097	.265

Means for groups in homogeneous subsets are displayed.

a. Uses Harmonic Mean Sample Size = 2.000.

encap

Tukey HSD

mins	N	Subset for alpha = 0.05				
		1	2	3	4	5
3	2	49.6000				
5	2		59.0000			
10	2			70.0000		
15	2				79.0000	
20	2					89.0000
25	2					95.0000
30	2					96.0000
Sig.		1.000	1.000	1.000	1.000	.067

Means for groups in homogeneous subsets are displayed.

a. Uses Harmonic Mean Sample Size = 2.000.

Table S7: Statistical analysis of one-way ANOVA and Post Hoc test on comparison of dishwashing performance of the formulated detergents A (contained free enzymes) and B (contained encapsulated enzymes) with commercial ADD, Finish®.

One-way ANOVA on data for Figure 5A & B

ANOVA

Finish (soft water)			Sum of Squares	Df	Mean Square	F	Sig.
Between Groups			4755.594	3	1585.198	279.761	.000
Within Groups			22.665	4	5.666		
Total			4778.259	7			
**Finish (hard water)**							
Between Groups	(Combined)		2431.735	3	810.578	377.892	.000
	Linear Term	Contrast	2126.216	1	2126.216	991.243	.000
		Deviation	305.519	2	152.759	71.217	.001
Within Groups			8.580	4	2.145		
Total			2440.315	7			

Comparison of formulated detergent with Finish ® (soft water)

Post hoc test for Figure 5A (21°C)

Multiple Comparisons

Dependent Variable: removal

Tukey HSD (21°c soft)

(I) type	(J) type	Mean Difference (I-J)	Std. Error	Sig.	95% Confidence Interval Lower Bound	Upper Bound
free	encapsulated	-1.00000	2.83373	.935	-12.8413	10.8413
	Finish	-29.20000*	2.83373	.004	-41.0413	-17.3587
encapsulated	free	1.00000	2.83373	.935	-10.8413	12.8413
	Finish	-28.20000*	2.83373	.004	-40.0413	-16.3587
Finish	free	29.20000*	2.83373	.004	17.3587	41.0413
	encapsulated	28.20000*	2.83373	.004	16.3587	40.0413

*. The mean difference is significant at the 0.05 level.

Post hoc test for Figure 5A (40°C)

Multiple Comparisons

Dependent Variable: removal

Tukey HSD (40 °c soft)

(I) type	(J) type	Mean Difference (I-J)	Std. Error	Sig.	95% Confidence Interval	
					Lower Bound	Upper Bound
Free	encapsulated	7.06500*	1.22113	.021	1.9623	12.1677
	Finish	-5.78500*	1.22113	.036	-10.8877	-.6823
encapsulated	free	-7.06500*	1.22113	.021	-12.1677	-1.9623
	Finish	-12.85000*	1.22113	.004	-17.9527	-7.7473
Finish	free	5.78500*	1.22113	.036	.6823	10.8877
	encapsulated	12.85000*	1.22113	.004	7.7473	17.9527

*. The mean difference is significant at the 0.05 level.

Post hoc test for Figure 5A (50°C)

Dependent Variable: removal

Tukey HSD (50°c soft water)

(I) type	(J) type	Mean Difference (I-J)	Std. Error	Sig.	95% Confidence Interval	
					Lower Bound	Upper Bound
free	encapsulated	-4.04000*	.79230	.029	-7.3508	-.7292
	Finish	-4.14000*	.79230	.028	-7.4508	-.8292
encapsulated	free	4.04000*	.79230	.029	.7292	7.3508
	Finish	-.10000	.79230	.991	-3.4108	3.2108
Finish	free	4.14000*	.79230	.028	.8292	7.4508
	encapsulated	.10000	.79230	.991	-3.2108	3.4108

*. The mean difference is significant at the 0.05 level.

Post hoc test for Figure 5A (60°C)

Dependent Variable: removal

Tukey HSD (60°c soft)

(I) type	(J) type	Mean Difference (I-J)	Std. Error	Sig.	95% Confidence Interval	
					Lower Bound	Upper Bound
Free	encapsulated	-4.28000	2.40312	.315	-14.3220	5.7620
	Finish	-4.95000	2.40312	.245	-14.9920	5.0920
encapsulated	free	4.28000	2.40312	.315	-5.7620	14.3220
	Finish	-.67000	2.40312	.959	-10.7120	9.3720
Finish	free	4.95000	2.40312	.245	-5.0920	14.9920
	encapsulated	.67000	2.40312	.959	-9.3720	10.7120

Post hoc test for Figure 5B (21°C)

Dependent Variable: removal

Tukey HSD 21 hard

(I) type	(J) type	Mean Difference (I-J)	Std. Error	Sig.	95% Confidence Interval	
					Lower Bound	Upper Bound
free	encapsulated	.03500	.84349	.999	-3.4897	3.5597
	Finish	-17.16500*	.84349	.001	-20.6897	-13.6403
encapsulated	free	-.03500	.84349	.999	-3.5597	3.4897
	Finish	-17.20000*	.84349	.001	-20.7247	-13.6753
Finish	free	17.16500*	.84349	.001	13.6403	20.6897
	encapsulated	17.20000*	.84349	.001	13.6753	20.7247

*. The mean difference is significant at the 0.05 level.

Post Hoc test for Figure 5B (40°C)

Dependent Variable: removal

Tukey HSD 40 hard

(I) type	(J) type	Mean Difference (I-J)	Std. Error	Sig.	95% Confidence Interval	
					Lower Bound	Upper Bound
free	encapsulated	-20.14000*	3.03535	.014	-32.8239	-7.4561
	Finish	-2.24000	3.03535	.761	-14.9239	10.4439
encapsulated	free	20.14000*	3.03535	.014	7.4561	32.8239
	Finish	17.90000*	3.03535	.020	5.2161	30.5839
Finish	free	2.24000	3.03535	.761	-10.4439	14.9239
	encapsulated	-17.90000*	3.03535	.020	-30.5839	-5.2161

*. The mean difference is significant at the 0.05 level.

Post Hoc test for Figure 5B (60°C)

Dependent Variable: removal

Tukey HSD50 hard

(I) type	(J) type	Mean Difference (I-J)	Std. Error	Sig.	95% Confidence Interval	
					Lower Bound	Upper Bound
free	encapsulated	-8.22500	5.18913	.376	-29.9089	13.4589
	Finish	-2.62500	5.18913	.874	-24.3089	19.0589
encapsulated	free	8.22500	5.18913	.376	-13.4589	29.9089
	Finish	5.60000	5.18913	.587	-16.0839	27.2839
Finish	free	2.62500	5.18913	.874	-19.0589	24.3089
	encapsulated	-5.60000	5.18913	.587	-27.2839	16.0839

*. The mean difference is significant at the 0.05 level.

Post Hoc test for Figure 5B (60°C)

Dependent Variable: removal

Tukey HSD (60 hard)

(I) type	(J) type	Mean Difference (I-J)	Std. Error	Sig.	95% Confidence Interval	
					Lower Bound	Upper Bound
free	encapsulated	-7.50000*	1.22474	.018	-12.6179	-2.3821
	Finish	-23.50000*	1.22474	.001	-28.6179	-18.3821
encapsulated	free	7.50000*	1.22474	.018	2.3821	12.6179
	Finish	-16.00000*	1.22474	.002	-21.1179	-10.8821
Finish	free	23.50000*	1.22474	.001	18.3821	28.6179
	encapsulated	16.00000*	1.22474	.002	10.8821	21.1179

*. The mean difference is significant at the 0.05 level.
